# Anthelmintic Efficacy of Gold Nanoparticles Derived from a Phytopathogenic Fungus, *Nigrospora oryzae*

**DOI:** 10.1371/journal.pone.0084693

**Published:** 2014-01-21

**Authors:** Pradip Kumar Kar, Sanatan Murmu, Saswati Saha, Veena Tandon, Krishnendu Acharya

**Affiliations:** 1 Parasitology Laboratory, Post Graduate Department of Zoology, Jhargram Raj College, Jhargram, West Midnapore, West Bengal, India; 2 Molecular and Applied Mycology and Plant Pathology Laboratory, Department of Botany, University of Calcutta, Kolkata, West Bengal, India; 3 Parasitology Laboratory, North-Eastern Hill University, Shillong, India; Virginia Commonwealth University, United States of America

## Abstract

Exploring a green chemistry approach, this study brings to the fore, the anthelmintic efficacy of gold nanoparticles, highlighting the plausible usage of myconanotechnology. Gold nanoparticles of ∼6 to ∼18 nm diameter were synthesized by treating the mycelia-free culture filtrate of the phytopathogenic fungus with gold chloride. Their size and morphology were confirmed by UV-Vis spectroscopy, DLS data, AFM and TEM images. The XRD studies reveal a crystalline nature of the nanoparticles, which are in cubic phase. The FTIR spectroscopic studies before and after the formation of nanoparticles show the presence of possible functional groups responsible for the bio-reduction and capping of the synthesized gold nanoparticles. The latter were tested as vermifugal agents against a model cestode *Raillietina* sp., an intestinal parasite of domestic fowl. Further, ultrastructural and biochemical parameters were used to corroborate the efficacy study.

## Introduction

Noble metal nanoparticles are centric to an emerging focus of nanoscience research, especially with respect to their properties, synthesis and applications. A plethora of physical, chemical and biological techniques continue to evolve leading to the production of noble metal nanoparticles [1], [2]. The integration of green chemistry principles to multidisciplinary nanoscience research has made scientists from different specializations, concerned about the need for developing environmentally benign and sustainable methods for synthesizing gold nanoparticles. Microorganisms being a group of highly diversified organisms found in nature, fit in quite appropriately to this requirement. Their high sustainability under ambient conditions of temperature, pressure and acidity, are highly preferred for the green synthesis of gold nanoparticles. Among diverse microorganisms, many bacteria, actinomycetes and fungi [3], [4] have been reported to synthesize gold nanoparticles.

Fungi, in particular, are a preferred choice for the purpose; their filamentous nature makes them withstand the flow pressure and agitation in a bioreactor and also due to their capability of accumulating metals by physicochemical and biological mechanisms. Furthermore, fungi are extremely efficient secretors of extra-cellular enzymes and are thus good candidates for their large-scale production. The cell-free culture filtrates of different fungi were used for biosynthesis of different nanoparticles like silver [5]–[7], selenium [8] and gold [9], [10].

Worldwide, traditional medicinal systems have taken advantage of the various useful natural products, which help in controlling or eradicating various types of helminth diseases, infecting both humans and cattle. A number of plants have been found to be useful in curing worm infections [11]–[14]. Results of *in vitro* tests with plant products against nematodes using methods such as motility and paralysis tests [15]–[18], egg hatch assays [19]–[21], and biochemical tests [22], [23] have been reported. Hordegen et al. screened several anthelmintic plant products against the larvae of gastrointestinal nematodes *Haemonchus contortus* [24]. Molgaard [25] screened the anthelmintic effect of extracts of 23 plant species against cysticercoids of the cestode, *Hymenolepis diminuta* and schistosomulae of the blood fluke, *Schistosoma* sp. (studied *in vitro*). Mohamed [26] also investigated the schistosomicidal properties of *Nigella sativa* seeds (*in vitro*) against *Schistosoma mansoni* miracidia, cercariae, and adult worms. Tuberostemonine, an alkaloid from *Stemona japonica* showed vermifugal effects on *H*. *diminuta* [27].

A large number of plants with medicinal properties have also been reported from India, too [28]–[30]. Roy et al. [31], [32] showed that the crude ethanol extract of the root peel of *Millettia pachycarpa* Benth (Leguminosae) were effective against *Raillietina echinobothrida*, the intestinal cestode parasite of domestic fowl. In another study, Tangpu and Temjenmogla [33] investigated the anticestodal efficacy of *Psidium guajava* L. leaf extract. *In vitro* anticestodal efficacies of folklore medicinal plants used by the Naga tribes of North-East India were also evaluated with promising results [34], [35]. There are also many studies on the efficacy of some putative anthelmintic plants against the trematode parasite, *Fasciolopsis buski* [36]. Tandon et al. [13] tested *in vitro* activity of root-tuber-peel extract of *Flemingia vestita*, an indigenous plant consumed by the natives in North-East India, against live model parasites (*Ascaris* spp. from pigs and humans, nematode and cestode infections from domestic fowl, and amphistomid trematodes from cattle).

Since time immemorial, colloidal gold has been used for medicinal purposes for various ailments [37], [38]. In Chinese traditional medicines, its use can be traced back to 2000 BC [39]. Red colloidal gold is still used in India as *Swarna Bhasma* (gold ash) as part of the Ayurvedic medicine, for rejuvenation and revitalization [40]. The major clinical uses of gold compounds are in treatment of rheumatic diseases, nephrotoxicity and cancer [41], [42].

In a previous study, Kar and Tandon [43] found that the tegumental and gastrodermal enzyme activity of *Fasciolopsis buski* declined following the treatment with crude extract of *Flemingia vestita* and its active component genistein. The current study aims to investigate the *in vitro* anthelmintic activity of the nanogold particles, synthesized by mycelia-free culture filtrate of the fungus *Nigrospora oryzae* treated with gold chloride, on worm parasites using a cestode (tapeworm) model. Alterations in the ultrastructure and biochemical attributes of the treated parasites versus their controls were substantiated in the present study.

## Materials and Methods

### Preparation of culture filtrates of the phytopathogen

*Nigrospora oryzae* (Strain Number: MAMP/C/77) was grown aerobically in liquid medium containing malt (0.3%), yeast extract (0.3%), peptone (0.5%) and autoclaved distilled water. Erlenmeyer flasks of 250 ml capacity were inoculated with fungal mycelia and incubated at 25–30°C with shaking at 150 rpm [7].

### Synthesis of gold nanoparticles from the culture filtrate

The mycelia-free culture filtrate was obtained by the separation of the full grown mycelial mat from the culture filtrate aseptically only after 8–9 days of the incubation period. The culture filtrate was then passed through Whatman filter paper No. 1 [7]. To 100 ml of the mycelia-free culture filtrate (MFCF), apposite amount of gold chloride (HAuCl_4_) was added to make the overall concentration of the salt to be 1 mM in the whole solution. Concurrently, a positive control (only MFCF without HAuCl_4_) and a negative control (only 100 ml of 1 mM HAuCl_4_ in de-ionized water) were also checked for comparison. All the above three sets were kept under constant agitation at room temperature in the dark. The formation of gold nanoparticles was preliminarily visualized by the change in color of the solution, which was further confirmed spectrophotometrically. The produced gold nanoparticles were separated out from the culture filtrate by centrifugation (at 12500 × g for 15 min) and the settled nanoparticles were washed thrice with de-ionized water. The washed gold nanoparticles were re-dispersed in water by ultrasonication (using Piezo-U-Sonic Ultrasonic Cleaner, Pus-60W).

### Characterization of the synthesized gold nanoparticles

The formation of gold nanoparticles was confirmed by the UV-Vis spectrophotometer (Hitachi 330 spectrophotometer) at 1 nm resolution only after the color change. The size of the nanoparticles was first measured by laser diffractometer (Zen 1600 Malvern USA) and then by Atomic Force Microscopy (AFM) using Nanoscope® 111A Veeco Multimode, USA. The characterization was done in tapping mode (NP10) with a silicon probe over scan sizes of 10 µm. The morphology of the nanoparticles was confirmed by Transmission Electron Microscopy (FP 5018/40, Tecnol G^2^ Spirit Bio TWIN). The XRD spectra were recorded from 30° to 80° 2*θ* angles using X-ray diffractometer (Seifert XDAL 3000) with CuK_α_ radiation operated at 45 kV and 30 mA. The FTIR (Fourier Transform Infrared) spectroscopy (Shimadzu FTIR 8400S) was used to obtain the information about the functional groups with which the nanoparticles were stabilized.

### Efficacy Testing

#### Treatments

The mature tapeworms (Raillietina sp.) were collected live from naturally infected domestic fowl (Gallus indicus), freshly slaughtered at local abattoirs, and incubated at 37±1°C in media containing varying dosages of the synthesized gold nanoparticles in ascending order, in Phosphate Buffered Saline (PBS). Praziquantel, a broad-spectrum anthelmintic, was used as the reference drug in appropriate dosages. A set of petri dishes having live worms in PBS, was maintained as control for each concentration. All the concentrations were tested with three replicates, each containing a batch of three worms with approximately the same size, weight and maturity. Time taken for the onset of paralysis and death of the parasites, was noted. The permanent immobilization of treated and control worms was determined visually when no motility occurred on physically disturbing them; death was confirmed by dipping the parasite in lukewarm water (40–50°C), which induced movements in the worm, still alive.

The incapacitated cestodes (after treatment with various test materials) were processed for further studies. Only the selected dosages of treatments were taken for the purpose of ultrastructure study and biochemical analyses; at these doses the onset of the paralytic state in the parasite could be attained in a relatively short span of time that compared well with the timings of the reference drug. Changes in the profile of tegument and gut-associated enzymes formed the basis of enzyme analysis.

Anthelmintic efficacy was determined in terms of motility, survivability, ultrastructural and biochemical changes, if any, in the treated worms.

#### Ultrastructure: Scanning Electron Microscopy (SEM)

The treated and control tapeworms were fixed in 10% neutral buffered formalin (NBF) or 3% Glutaraldehyde at 4°C for 24 h, washed in PBS and dehydrated with ascending grades of acetone to pure dried acetone. The specimens were then critical-point-dried using liquid carbon dioxide as the transitional fluid or treated with tetramethylsilane (TMS–[CCH3]4Si, boiling point 26.3°C, surface tension 10.3 dynes/cm at 20°C) for 15 min and air dried at 25°C, following the method of Dey et al. [44]. The dried material was put on a metal stub according to the orientation required and sputter coated with gold in a fine-coat ion sputter, JFC-1100 (JEOL). The gold-coated specimens were observed using a Philips SEM (model no. LEO 435 VP 501B) at electron accelerating voltage ranging between 10–20 kilovolt (kV).

#### Biochemical Assays

Acid Phosphatase (AcPase) and Alkaline Phosphatase (AlkPase) : Assays for AcPase and AlkPase activities were done by estimating the p-nitrophenol product following the method of Plummer (1988) with necessary modification in the concentration of the buffer and substrate [45]. One unit of the enzyme activity is defined as that amount which catalyzed the formation of 1 mM of p-nitrophenol/h at 37±1°C.

Adenosine triphosphatase (ATPase): Following the method of Kaplan with Na-ATP as the substrate [46], activity of ATPase was assayed by estimating the free phosphate released. One unit of ATPase is defined as the amount which catalyzed the release of 1 µmole of phosphate / h at 37±1°C from ATP.

5′-Nucleotidase (5′-Nu): The enzyme activity was assayed by estimating the free phosphate released following the method of Bunitian using AMP as the substrate [47]. One unit of 5′-Nu activity is defined as that amount which catalyzed the release of l µmole of phosphate/h at 37±1°C from AMP.

Protein: The protein content was estimated following the method of Lowry et al. [48] using bovine serum albumin as a standard.

All chemicals used in the present study were procured from Sigma Chemicals, USA or SRL, India.

## Results

### UV-Vis spectral analysis

**Figure 1:**
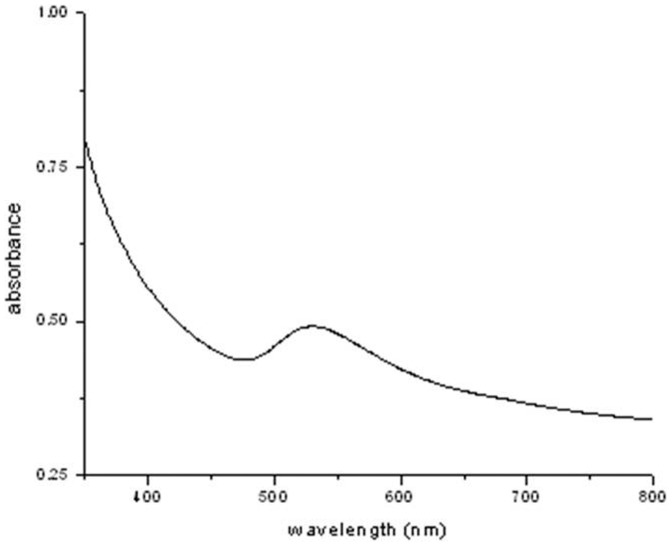
UV-Vis spectra of synthesized gold nanoparticles.

Gold nanoparticles having their unique and tunable surface plasmon resonance (SPR) property have been considered in many applications of biomedical sciences. The optical absorption spectrum of the metal nanoparticles is sensitive to several factors like size, shape, particle-particle interaction with the medium and local refractive index [49]. Moreover, due to the fact that the color of colloidal gold is attributed to specific SPR arising due to the collective oscillations of free conduction electrons induced by an interacting electromagnetic field, the formation of nanoparticles was established by UV-Vis spectroscopy [50]. These nanoparticles showed a sharp peak at 550 nm (visible red region) as shown in Fig. 1. The reduction of gold ions from Au (III) to Au (0) state and simultaneous formation of gold nanoparticles was detected (on addition of HAuCl_4_ to MFCF) preliminarily by the change in color from light yellow to bluish red to purple within 3 h of addition of the gold salt. No such color change was observed in the positive control (MFCF without HAuCl_4_) and negative control (1 mM solution of HAuCl_4_ in de-ionized water) sets.

### Morphological analysis

**Figure 2:**
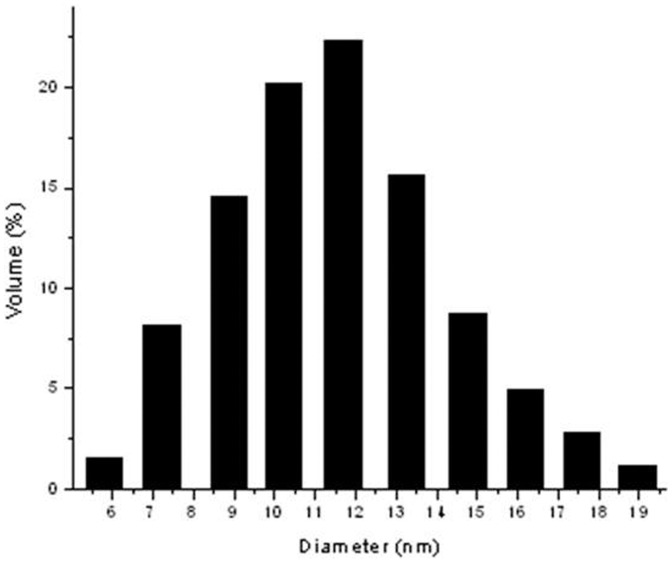
DLS data of the gold nanoparticles.

**Figure 3:**
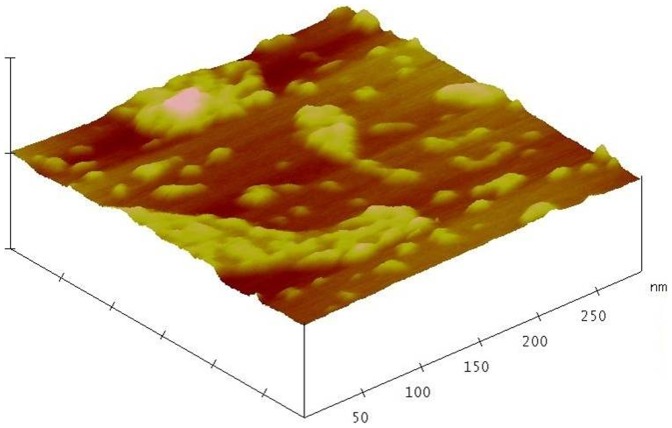
AFM image 3D view of gold nanoparticles.

**Figure 4:**
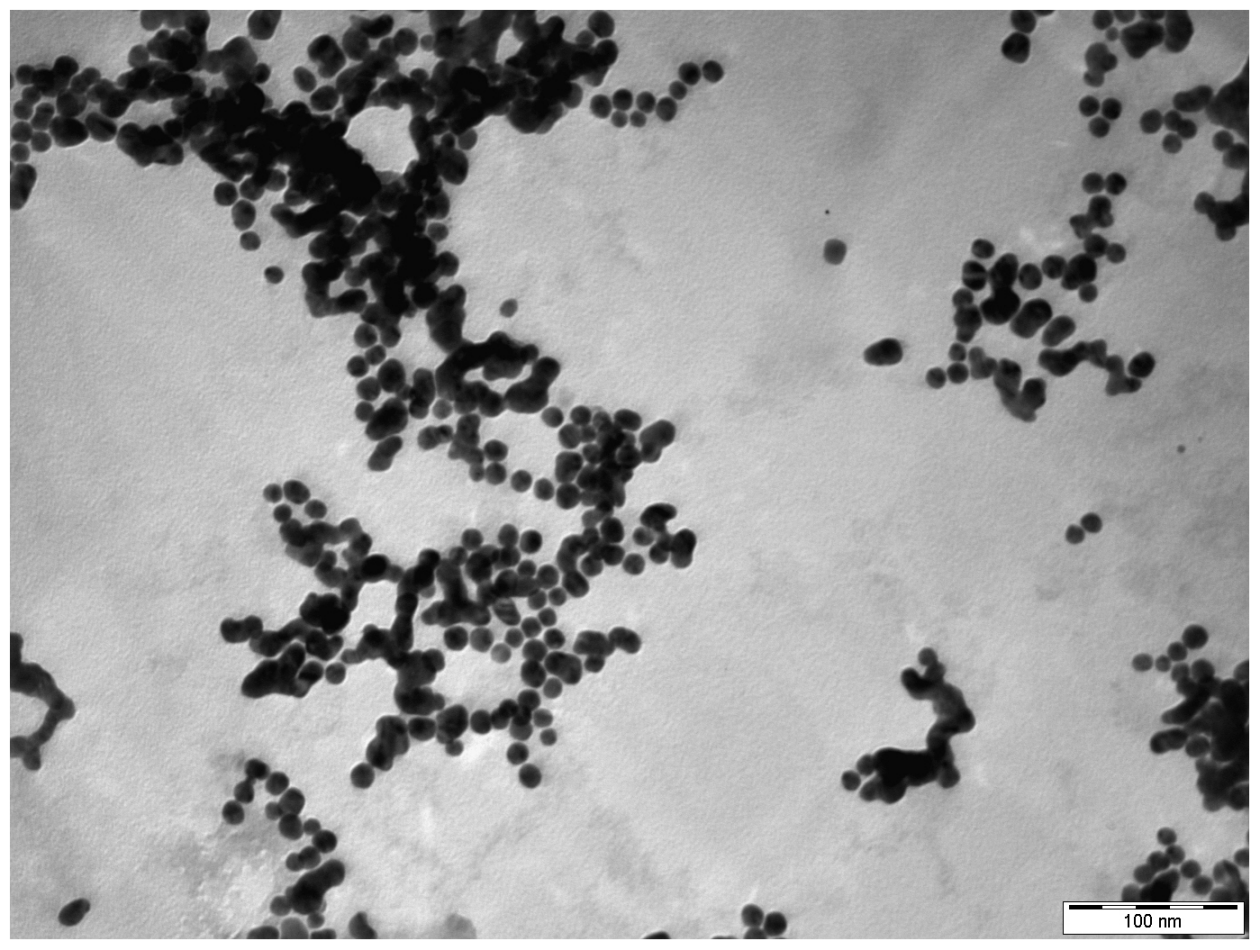
TEM image of gold nanoparticles.

The size of the synthesized gold nanoparticles, formerly determined by laser diffractometer showed a range of ∼6 nm to ∼18 nm (Fig. 2). Further confirmation was done by AFM (Fig. 3) and TEM (Fig. 4) studies, which reveal the monodispersed spherical nature of the bio-reduced gold nanoparticles.

### XRD analysis

**Figure 5:**
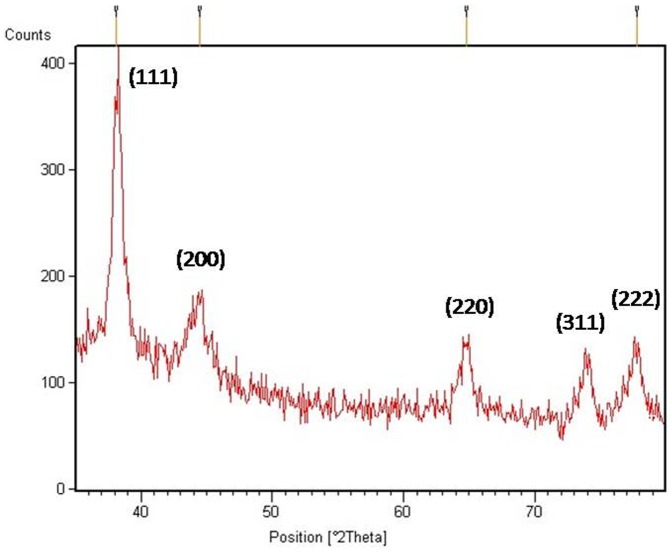
XRD data showing 5 peaks representing different facets of face-centered cubic gold nanoparticles.

XRD analyses were performed to confirm the monocrystalline nature of the gold nanoparticles (Fig 5). Dried and powdered samples of the synthesized nanoparticles showed five diffraction peaks obtained in the 2*θ* range of 30° to 80° corresponding to *[111]*, *[200]*, *[220]*, *[311]* and *[222]*, indicating that the precipitate is composed of pure crystalline gold (International Center for Diffraction Data, ICDD No 4-0783). As per the XRD pattern, a very intense Brag reflection for the *[111]* lattice is observed suggesting the gold nanoparticles are lying flat on a planar surface.

### FTIR analysis

**Figure 6:**
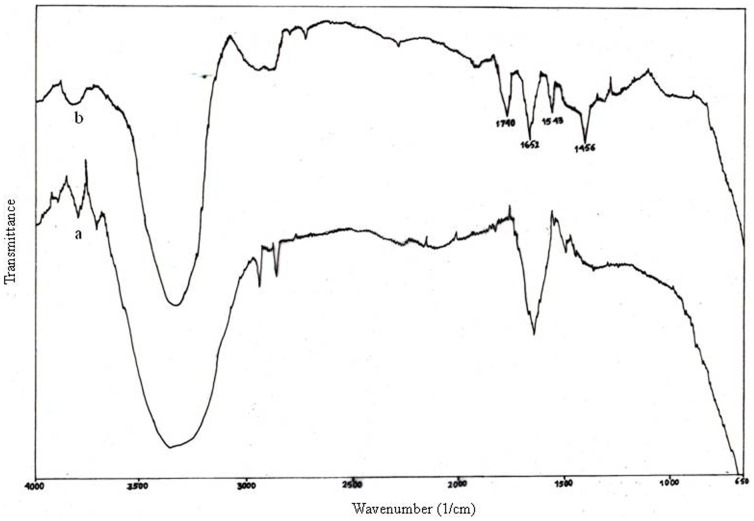
FTIR spectra a) before and b) after reduction of the gold salt.

FTIR measurements (Fig. 6) were carried out to verify the possible interaction between the gold ions and the functional groups of biomolecules present in the MFCF responsible for the reduction and stabilization of the synthesized nanoparticles [9]. The strong band at 3300–3500 cm^-1^ indicates a strong hydrogen bonding. Compared to the FTIR bands of the fungal culture filtrate (kept as a control), after reduction of gold salt, strong peaks at 1652 cm^-1^ and 1543 cm^-1^ were found representing the presence of amide I and amide II, respectively, which may be due to the carbonyl stretch and N-H stretch vibrations of amide linkages of protein residues. The band at 1456 cm^-1^ may be assigned to methylene scissoring vibrations from the proteins/biomolecules in the solutions. Another strong band at 1740 cm^-1^ may correspond to the carbonyl stretch vibrations of ketones, aldehydes and carboxylic acids, those which are noteworthy for the reduction of gold ions in the fungal culture filtrate with the help of extracellularly secreted fungal enzymes/biomolecules during their growth in the medium.

### Efficacy Testing

**Figure 7:**
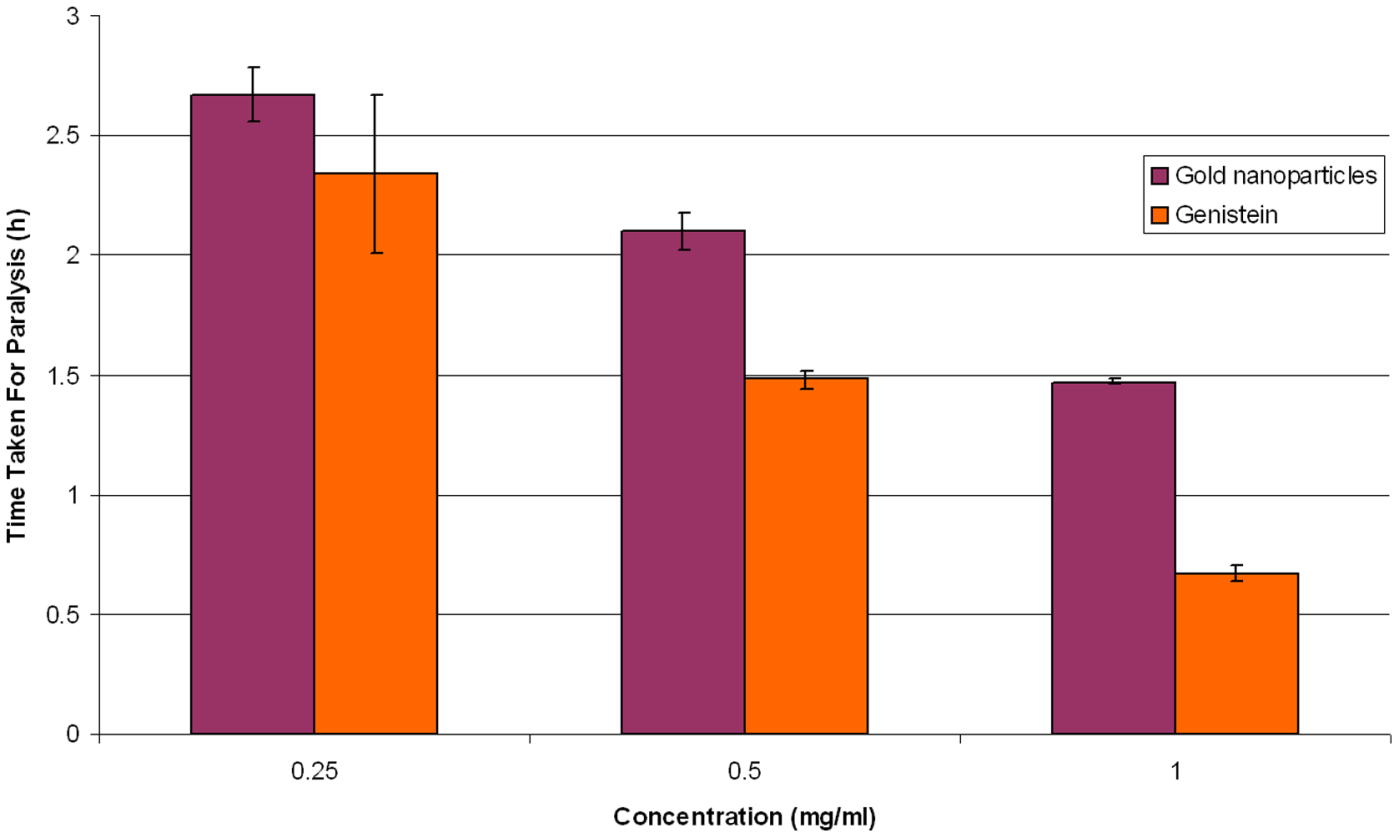
Results of nanogold efficacy test against the cestode *Raillietina* sp.

The *in vitro* testing of the efficacy of gold nanoparticles against the cestode parasite showed a paralysis time of 2.67 h, 2.1 h, 1.47 h and death time of 3.22 h, 2.83 h, 2.55 h for dosages of 0.25 mg/ml, 0.5 mg/ml, 1.0 mg/ml, respectively. The most efficacious dose was found to be 1.0 mg/ml, wherein the onset of paralysis of parasites occurred after 1.47 h and death after 2.55 h of treatment. A similar treatment of the parasite with genistein showed a paralysis time of 2.34 h, 1.48 h, 0.67 h and death time of 3.83 h, 2.13 h, 1.51 h for dosages of 0.25 mg/ml, 0.5 mg/ml, 1.0 mg/ml, respectively. (Table 1 and Fig. 7). The parasite kept at 37±1°C in PBS only without the addition of nanogold particles or genistein survived for 72±0.05 h.

**Table 1:** Results of nanogold efficacy test against the cestode *Raillietina* sp.

Treatment: Concentration (mg/ml)	Time (h) taken for paralysis (P) and death (D) of the worm *Raillietina* sp. post incubation*	
	P	D
Gold nanoparticles:		
0.25	2.67 ± 0.11	3.22 ± 0.06
0.5	2.1 ± 0.08	2.83 ± 0.05
1.0	1.47 ± 0.01	2.55 ± 0.14
Genistein:		
0.25	2.34 ± 0.33	3.83 ± 0.52
0.5	1.48 ± 0.04	2.13 ± 0.13
1.0	0.67 ± 0.031	1.51 ± 0.04

*Data represent mean values ± SD of three experiments. Student's *t*-test insignificant. Worms incubated in control medium showed physical activity till 72±0.05 h.

Culture filtrate of gold nanoparticles had no effect on the model cestodes.

### Ultrastructural studies

**Figure 8:**
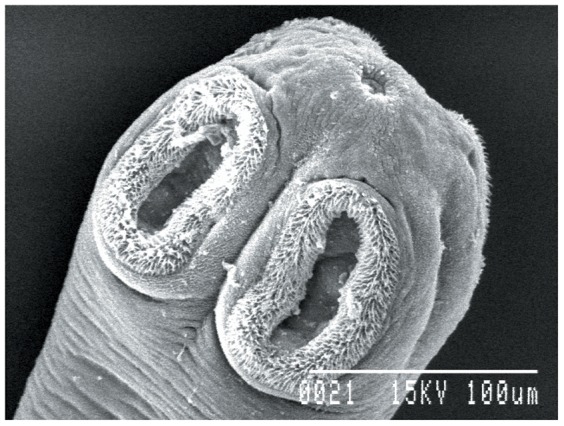
Ultrastructure of scolex of *Raillietina* sp. 500× (Control).

**Figure 9:**
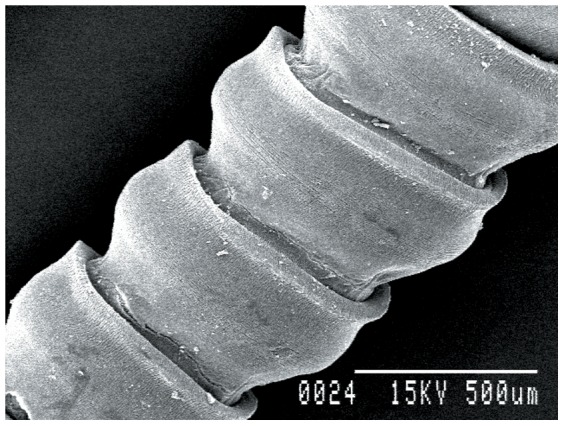
Ultrastructure of proglottid of *Raillietina* sp. 80× (Control).

**Figure 10:**
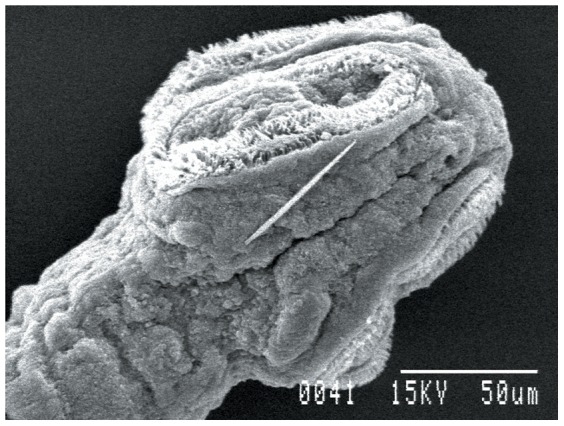
*Raillietina* sp. worms treated with gold nanoparticles, scolex region 600×.

**Figure 11:**
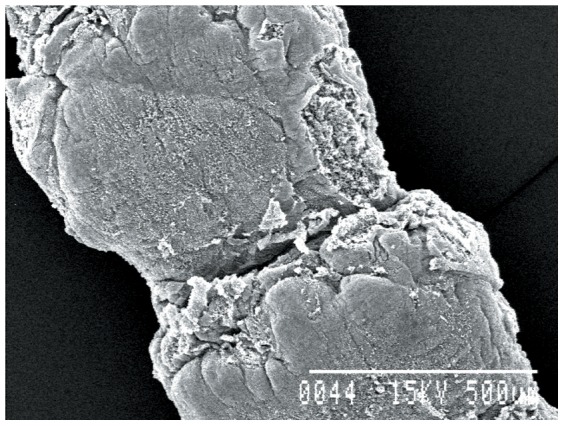
Gravid proglottids of *Raillietina* sp. worms after treatment with gold nanoparticles 100×.

**Figure 12:**
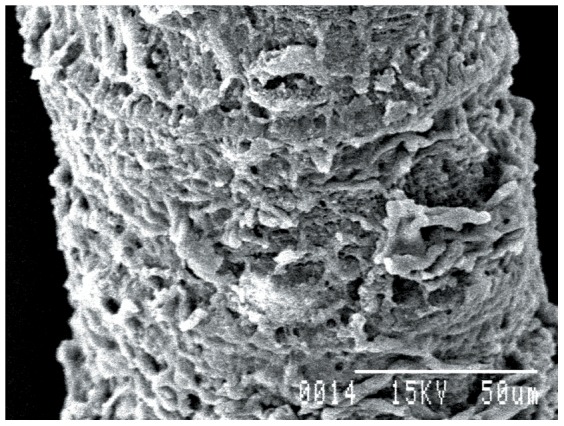
A single proglottid of *Raillietina* sp. after treatment with gold nanoparticles 800×.

Worms treated with 1.0 mg/ml dosage of synthesized gold nanoparticles showed significant changes as compared to controls. The sucker region of the cestode showed a natural contour in the control worm, while in the treated worms marked changes in the ultrastructural morphology were observed (Figs. 8–10). Stereoscan observation of the treated parasites revealed disorganization of the tegumental architecture showing major damages in the form of wrinkles, lesions and ruptures on the surface tegument of the parasite; the microtriches showed a clustered appearance all over the body surface (Figs. 11, 12).

### Enzyme Analysis

The results of enzyme assays are described in Table 2. The AcPase activity in *Raillietina* sp. paralyzed with the test materials (gold nanoparticles and genistein) was inhibited by 29.16% and 34.72%, respectively, whereas Praziquantel caused a decline by 48.61% compared to the controls. A varying degree of inhibition of the AlkPase activity (37.02–44.65%) in comparison to control was observed after treatment with the plant-derived components. The ATPase activity also got reduced by 24.28% and 55.94% in treatments with gold nanoparticles and genistein, respectively, while in the reference drug-treated parasite a decrease by 44.97% was recorded. Biochemical analysis of 5′-Nu showed a decrease in the enzyme activity by 37.85% for gold nanoparticles and 49.62% for genistein. The parasites treated with the reference drug showed a decrease by 41.18% (Table 2).

**Table 2:** Biochemical effect on tegumental enzymes of *Raillietina* sp.

Treatment (mg/ml)	Enzyme activity (total^a^ / specific^b^)				% change			
	AcP (A)	AlkP(K)	ATP (T)	5′Nu (5′)	A	K	T	5′
Control (in 0.9% PBS)	7.2±0.3 / 1.2±0.02	26.2±3.6 / 3.3±0.76	456.16±71.4 / 49±2.5	39.1±2.3 / 4.37±0.54	-	-	-	-
Gold nanoparticles (20)	5.1±2.3 / 1.2±0.01	16.5±3.5 / 3.5±0.9	345.4±69 / 45.4±5	24.3±2.4 / 3.8±1.1	29.16	37.02	24.28	37.85
Genistein (0.5)	4.7±0.11 / 1.2±0.2	12.8±1.8 / 3.7±0.81	201±33 / 48±5.5	19.7±1.4 / 4.6±0.4	34.72	51.15	55.94	49.62
Praziquantel (0.01)	3.7±0.13 / 1.1±0.07	14.5±2.6 / 2.7±0.9	251±34.1 / 48±4.1	23±2.3 / 4.5±0.8	48.61	44.65	44.97	41.18

Values are given as mean (± S.E.) from five replicate assays.

^a^ Enzyme activity expressed as an unit which consumes 1.0 µmol substrate/g wet wt. tissue/h.

^b^ Specific activity expressed as unit/mg protein/h.

p value significant at < 0.05.

## Discussion

In the present study a cost-effective, simple and rapid approach to form gold nanoparticles and their anthelmintic efficacy against a model parasite has been demonstrated. Nanogold particles have been used for a longtime for delivery of drug molecules into cells and large biomolecules [51]–[53]. Recent investigations have revealed that gold nanoparticles have a strong potential to fight against a wide range of cancer cells and induce apoptosis [54]. Here, we unveil a novel approach towards the application of gold nanoparticles as a vermifugal agent. The tegument in flatworms has an absorptive potential. The processes of secretion and absorption appear to operate concurrently in the tegument of most cestodes, although one or the other function may predominate at any given time [55]. The current study substantiates the fact that the gold nanoparticles (6–18 nm diameter) produced by bioreduction of chloroauric acid using mycelia-free culture filtrate, have a detrimental effect on the ultrastructure of *Raillietina* sp.; the disorientation of microtriches, spines and scales was noted along with distortion and disfigurement of the tegument, breakage and sloughing off of the tegumental surface structures. It is noteworthy to mention that the culture filtrate had no effect on *Raillietina* sp. Cestodes are soft-bodied parasites and the tegument is their only interface, through which various physiological functions such as digestion and absorption takes place. The gold nanoparticles seem to affect this normal physiological functioning causing paralysis and subsequent death of the cestode, as has been described earlier in the case of *Raillietina echinobothrida* and *Fasciolopsis buski* (the giant intestinal fluke) on being treated with the crude extract of *Flemingia vestita* and its bioactive component, genistein [13], [56]. Ultrastructural changes in the tegument are linked to a possible action of the drug as an inhibitor of protein synthesis [57]. Changes in the tegumental architecture on treatment with gold nanoparticles suggest that the phytopathogenic fungal products bring about permeability changes in the tegument of the cestodes. In a number of helminth parasites AcPase, AlkPase, ATPase and 5′-Nu are known to be closely associated with the tegument, subtegument, somatic musculature and gut [58]–[61]. As revealed in the present *in vitro* study, alterations occurred in the enzyme activity of the parasite after treatment with gold nanoparticles, in synchrony with the effectiveness of the reference botanicals and the reference drug tested in previous studies. Further *in vivo* studies are required on the anthelmintic efficacy of nanogold particles, to corroborate and augment the *in vitro* study results and pave the way towards a smooth transition to commercially viable therapeutics. Currently, studies on anthelmintics derived from natural sources have been an important field of research. The present study is the first of its kind to explore the effectiveness of gold nanoparticles (derived from phytopathogenic fungus) in a plausible anthelmintic role.

## References

[1] Mohanpuria P, Rana NK, Yadav SK (2008) Biosynthesis of nanoparticles: technological concepts and future applications. J Nanopart Res 10: 507–517.

[2] Luechinger NA, Grass RN, Athanassiou EK, Stark WJ (2010) Bottom-up fabrication of metal/metal nanocomposites from nanoparticles of immiscible metals. Chem Mat 22: 155–160.

[3] Tikariha S, Singh S, Banerjee S, Vidyarthi AS (2012) Biosynthesis of gold nanoparticles, scope and applications: a review. Int J Pharm Res 3(6): 1603–1615.

[4] Acharya K, Sarkar J, Deo SS (2009) Mycosynthesis of nanoparticles. In Bhowmik PK, Basu SK, Goyal A, editors. Advances in Biotechnology, Oak Park: Bentham Science Publishers Ltd, USA. 204–215.

[5] Saha S, Sarkar J, Chattopadhyay D, Patra S, Chakraborty A, et al (2010) Production of silver nanoparticles by a phytopathogenic fungus *Bipolaris nodulosa* and its antimicrobial activity. Dig J Nanomater Biostruct 5: 887–895.

[6] Sarkar J, Chattopadhyay D, Patra S, Deo SS, Sinha S, et al (2011a) *Alternaria alternata* mediated synthesis of protein capped silver nanoparticles and their genotoxic activity. Dig J Nanomat Biostruct 6: 563–573.

[7] Saha S, Chattopadhyay D, Acharya K (2011) Preparation of silver nanoparticles by bio-reduction using *Nigrospora oryzae* culture filtrate and its antimicrobial activity. Dig J Nanomat Biostruct 6: 1526–1535.

[8] Sarkar J, Dey P, Saha S, Acharya K (2011b) Mycosynthesis of selenium nanoparticles. Micro Nano Lett 6: 599–602.

[9] Sarkar J, Ray S, Chattopadhyay D, Laskar A, Acharya K (2012) Mycogenesis of gold nanoparticles using a phytopathogen *Alternaria alternata* . Bioproc Biosyst Eng 35: 637–643. 10.1007/s00449-011-0646-4 22009439

[10] Sarkar J, Ray SK, Laskar A, Chattopadhyay D, Acharya K (2013) Bioreduction of chloroaurate ions to gold nanoparticles by culture filtrate of *Pleurotus sapidus* . Mater Lett 92: 313–316.

[11] Perry LM (1980) Medicinal plants of East and Southeast Asia. London: MIT Press.

[12] Chhabra SC, Mahunnah RLA, Mshiu EN (1990) Plants used in traditional medicine in Eastern Tanzania (Mimosaceae to Papilionaceae). J Ethnopharmacol 29(3): 295–323. 2214816 10.1016/0378-8741(90)90041-q

[13] Tandon V, Pal P, Roy B (1997) *In vitro* anthelmintic activity of root-tuber extract of *Flemingia vestita*, an indigenous plant in Shillong, India. Parasitol Res 83: 492–498. 9197399 10.1007/s004360050286

[14] Biftu D, Nurfeta A, Jobre Y (2004) Evaluation of anthelmintic activities of crude leaf extracts of three indigenous herbal plants against ovine gastrointestinal nematodosis. Ethiop Vet J 8(2): 57–65.

[15] Robinson RD, Williams LA, Lindo JF, Terry SI, Mansingh A (1990) Inactivation of *Strongyloides stercoralis* filariform larvae *in vitro* by six Jamaican plant extracts and three commercial anthelmintics. W Indian Med J 39(4): 213–217. 2082565

[16] Perett S, Whitfield PJ (1995) Aqueous degradation of isoflavonoides in an extract of *Milettia thonningii* (leguminoceae) which is larvicidal towards schistosomes. Phytother Res 9(6): 401–404.

[17] Kaushik RK, Katiyar JC, Sen AB (1981) A new *in vitro* screening technique for anthelmintic activity using *Ascaridia galli* as a test parasite. Indian J Anim Sci 5: 869–872.

[18] Parveen N (1991) Antifilarial activity of *Vitex negundo* against *Setaria cervi* . Fitoterapia 62: 163–165.

[19] Ketzis JK, Taylor A, Bowman DD, Brown DL, Warnick LD, et al (2002) *Chenopodium ambrosioides* and its essential oil as treatments for *Haemonchus contortus* and mixed adult-nematode infections in goats. Small Ruminant Res 44: 193–200.

[20] Pessoa LM, Morais SM, Bevilaqua CML, Luciano JHS (2002) Anthelmintic activity of essential oil of *Ocimum gratissimum* Linn. and eugenol against *Haemonchus contortus* . Vet Parasitol 109: 59–63. 12383625 10.1016/s0304-4017(02)00253-4

[21] Alawa CBI, Adamu AM, Gefu JO, Ajanusi OJ, Abdu PA, et al (2003) *In vitro* screening of two Nigerian medicinal plants (*Vernonia amygdalina* and *Annona senegalensis*) for anthelmintic activity. Vet Parasitol 113: 73–81. 12651218 10.1016/s0304-4017(03)00040-2

[22] Kumar D, Mishra SK, Tandan SK, Tripathi HC (1995) Possible mechanism of anthelmintic action of palasonin on *Ascaridia galli* . Indian J Pharmacol 27: 161–166.

[23] Khunkitti W, Fujimaki Y, Aoki Y (2000) *In vitro* antifilarial activity of extracts of the medicinal plant *Cardiospermum halicacabum* against *Brugia pahangi* . J Helminthol 74: 241–246. 10953224 10.1017/s0022149x00000342

[24] Hordegen P, Cabaret JH, Hertzberg A, Langhans WC, Maurer V (2006) *In vitro* screening of six anthelmintic plant products against larval *Haemonchus contortus* with a modified methyl-thiazolyl-tetrazolium reduction assay. J Ethnopharmacol 108: 85–89. 16725288 10.1016/j.jep.2006.04.013

[25] Molgaard P, Nielsen SB, Rasmussen DE, Drummond RB, Makaza N, et al (2001) Antihelmintic screening of Zimbabwean plants traditional used against schistosomiasis. J Ethnopharmacol 74: 257–264. 11274827 10.1016/s0378-8741(00)00377-9

[26] Mohamed AM, Metwally NM, Mahmoud SS (2005) *Sativa* seeds against *Schistosoma mansoni* different stages. Mem Inst Oswaldo Cruz Vol. 100. 10.1590/s0074-02762005000200016 16021310

[27] Terada M, Sano M, Ishii AI, Kino H, Fukushima S, et al (1982) Studies on chemotherapy of parasitic helminths (III). Effects of tuberostemonine from *Stemona japonica* on the motility of parasitic helminths and isolated host issues. Nippon Yakurigaku Zasshi 79(2): 93–103. 7200048 10.1254/fpj.79.93

[28] Bhakuni DS, Goel AK, Jain S, Mehrotra BN, Patnaik GK, et al (1988) Screening of Indian plants for biological activity: Part XIII. Ind J Exp Biol 26(11): 883–904. 3248849

[29] Shilaskar DV, Parasher GC (1989) Evolution of indigenous anthelmintics. *In vitro* screening of some indigenous plants for their anthelmintic activity against *Ascaridia galli* . Indian J Indigenous Med 6: 49–53.

[30] Pal P, Tandon V (1998) Anthelmintic efficacy of *Flemingia vestita* (Leguminosae): Genistein-induced alterations in the activity of tegumental enzymes in the cestode, *Raillietina echinobothrida* . Parasitol Int 47(1): 233–243.

[31] Roy B, Lalchhandama K, Dutta BK (2008a) Scanning electron microscopic observations on the *in vitro* anthelmintic effects of *Millettia pachycarpa* on *Raillietina echinobothrida* . Pharmacogn Mag 4(13): 20–26.

[32] Roy B, Dasgupta S, Tandon V (2008b) Ultrastructural observations on tegumental surface of *Raillietina echinobothrida* and its alterations caused by root-peel extract of *Millettia pachycarpa.* . Microsc Res Techniq 71(11): 810–815. 10.1002/jemt.20623 18767049

[33] Tangpu V, Temgenmogla A, Yadav AK (2006) Anticestodal efficacy of *Psidium guajava* against experimental *Hymenolepis diminuta* infection in rats. Indian J Pharmacol 38(1): 29–32.

[34] Temjenmongla A, Yadav AK (2005) Anticestodal efficacy of folklore medicinal plants of Naga tribes in north-east India. Afr J Tradit Complement Altern Med 2: 129–133.

[35] Yadav AK, Tangpu V (2006) *In vitro* anticestodal evaluation of some medicinal plants used by the Naga traditional healers. Pharmacologyonline 3: 90–95.

[36] Tandon V, Lyndem LM, Kar PK, Pal P, Das B, et al (2004) Anthelmintic efficacy of rhizome-pulp extract of *Stephania glabra* and aerial root extract of *Trichosanthes multiloba* in vitro: two indigenous plants in Shillong, India. J Parasit Dis 28(1): 37–44.

[37] Kamei H, Koide T, Kojima T, Hashimoto Y, Hasegawa M (1998) Effect of gold on survival of tumorbearing mice. Cancer Biother Radiopharm 1998 13: 403–406. 10.1089/cbr.1998.13.403 10851432

[38] Bajaj S, Vohora SB (1998) Analgesic activity of gold preparations used in Ayurveda & Unani-Tibb. Indian J Med Res 108: 104–111. 9798337

[39] Daniel MC, Astruc D (2004) Gold nanoparticles: assembly, supramolecular chemistry, quantumsize-related properties, and applications toward biology, catalysis, and nanotechnology. Chem Rev 104: 293–346. 14719978 10.1021/cr030698+

[40] Hillyer JF, Albrecht RM (2001) Gastrointestinal persorption and tissue distribution of differently sized colloidal gold nanoparticles. J Pharm Sci 90: 1927–1936. 11745751 10.1002/jps.1143

[41] Richards DG, McMillin DL, Mein EA, Nelson CD (2002) Gold and its relationship to neurological / glandular conditions. Int J Neurosci 112: 31–53. 12152404 10.1080/00207450212018

[42] Mukherjee P, Bhattacharya R, Bone N, Lee YK, Patra CR, et al (2007) Potential therapeutic application of gold nanoparticles in B-chronic lymphocytic leukemia (BCLL): enhancing apoptosis. J Nanobiotechnol 5: 4 doi:10.1186/1477-3155-5-4 10.1186/1477-3155-5-4 PMC1876244 17488514

[43] Kar PK, Tandon V (2004) Anthelmintic efficacy of genistein, the active principle of *Flemingia vestita* (Fabaceae): Alterations in the activity of the enzymes associated with the tegumental and gastrodermal interfaces of the trematode, *Fasciolopsis buski* . J Parasit Dis 28(1): 45–56.

[44] Dey S, Basu Baul TS, Roy B, Dey D (1989) A new rapid method of air-drying for scannnig electron microscopy using tetramethylsilane. J Microscopy 156: 259–261.

[45] Plummer DT (1988) An introduction to practical biochemistry. Tata-McGraw Hill, New Delhi.

[46] Kaplan C (1957) Methods in Enzymology. Vol. III. Academic Press, New York. Fiske, CH and Subba Rao, Y (1925). The colorimetric determination of phosphorus. J Biol Chem 66: 375–400.

[47] Bunitian HC (1970) Deamination of nucleotides and the role of their deamino forms in ammonia formation from amino acids. In Lajtha A, Handbook of Neurochemistry, Plenum, New York.

[48] Lowry OH, Rosebrough NJ, Farz AL, Randall RJ (1951) Protein measurement with the folin phenol reagent. J Biol Chem 193: 265–275. 14907713

[49] Verma VC, Singh SK, Solanki R, Prakash S (2011) Biofabrication of anisotropic gold nanotriangles using extract of endophytic *Aspergillus clavatus* as a dual functional reductant and stabilizer. Nano Res Let 6: 16–23. 10.1007/s11671-010-9743-6 PMC3211211 27502640

[50] Mulvancy P (1996) Surface plasmon spectroscopy of nano sized metal particles. Langmuir 12: 788–800.

[51] Chen PC, Mwakari SC, Oyelere AK (2008) Gold nanoparticles: from nanomedicine to nanosensing. Nanotechnol Sci Appl 1: 45–66. 24198460 10.2147/nsa.s3707 PMC3781743

[52] Sperling RA, Gil PR, Zhang F, Zanella M, Parak WJ (2008) Biological application of gold nanoparticles. Chem Soc Rev 37: 1896–1908. 18762838 10.1039/b712170a

[53] Ghosh P, Han G, De M, Kim KC, Rotello MV (2008) Gold nanoparticles in delivery applications. Adv Drug Deliv Rev 60: 1307–1315. 18555555 10.1016/j.addr.2008.03.016

[54] Tikhira S, Singh S, Banerjee S, Vidhyarthi AS (2012) Biosynthesis of gold nanoparticles, scope and application: A review. Int J Pharm Sci Res 3(6): 1603–1615.

[55] Roy B, Dasgupta S, Tandon V (2008) Ultrastructural observations on tegumental surface of *Raillietina echinobothrida* and its alterations caused by root-peel extract of *Millettia pachycarpa.* . Microsc Res Techniq 71(11): 810–815. 10.1002/jemt.20623 18767049

[56] Roy B, Tandon V (1996) Effect of root tuber peel extract of *Flemingia vestita*, a leguminous plant, on *Artyfechinostomum sufrartyfex* and *Fasciolopsis buski*: a scanning electron microscopy study. Parasitol Res 82(3): 248–252. 8801558 10.1007/s004360050104

[57] Anderson HR, Fairweather I (1995) *Fasciola hepatica*: ultrastructural changes to the tegument of juvenile flukes following incubation in vitro with the deacetylated (amine) metabolite of diamphencthide. Int J Parasitol 25(3): 319–333. 7601590 10.1016/0020-7519(94)00105-w

[58] Pappas PW (1988) Acid phosphatase activity in the isolated brush border membrane of the tapeworm, *Hymenolepis diminuta*: Partial characterization and differentiation from the alkaline phosphatase activity. J Cell Biochem 37(4): 395–403. 3417789 10.1002/jcb.240370407

[59] Leon P, Monteoliva M, Sanchez-Moreno M (1989) Isoenzyme patterns of phosphatases and esterases in *Fasciola hepatica* and *Dicrocoelium dendriticum* . Vet Parasitol 30: 297–304. 2728320 10.1016/0304-4017(89)90099-x

[60] Kwak KH, Kim CH (1996) Characteristics of alkaline and acid phosphatase in *Spirometra erinacei* . Korean J Parasitol 34(1): 69–77. 8820743 10.3347/kjp.1996.34.1.69

[61] Fetterer RH, Rhoads ML (2000) Characterization of acid phosphatase and phosphorylcholine hydrolase in adult *Haemonchus contortus* . J Parasitol 86(1): 1–6. 10701555 10.1645/0022-3395(2000)086[0001:COAPAP]2.0.CO;2

